# Supplementary material for: Presenilin Deficiency Results in Cellular Cholesterol Accumulation by Impairment of Protein Glycosylation and NPC1 Function
Source: Int J Mol Sci. 2024 May 16;25(10):5417. doi: 10.3390/ijms25105417 (PMC11121565; doi:10.3390/ijms25105417)
Supplement: Supplementary file 1 [file ijms-25-05417-s001.zip › ijms-2984698-supplementary.pdf]

Supplementary Figure. S1

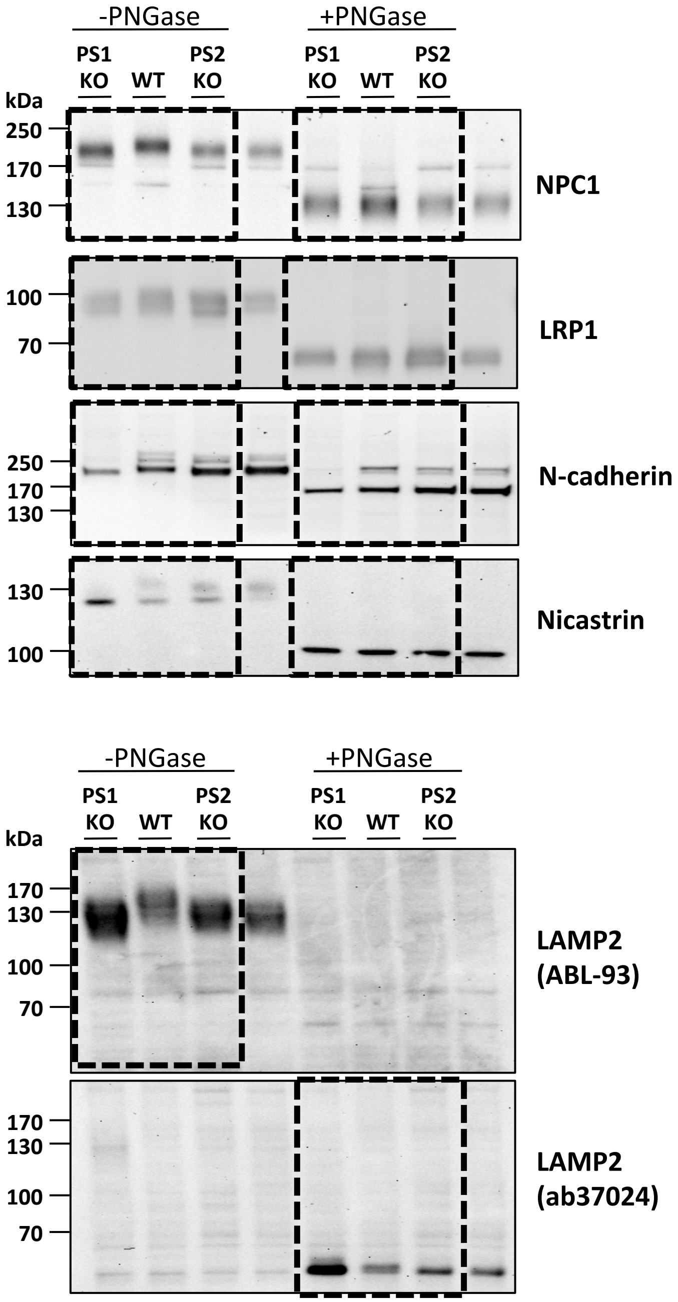

Supplementary Figure. S1. The original western blots of figure 4A. The framed area by rectangles are shown in Fig. 4A.

Supplementary Figure. S2

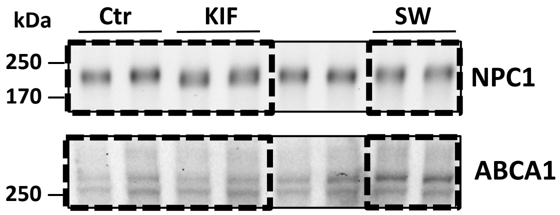

Supplementary Figure. S2. The original western blots of figure 6C. The framed area by rectangles are shown in Fig. 6C.

Supplementary Figure. S3

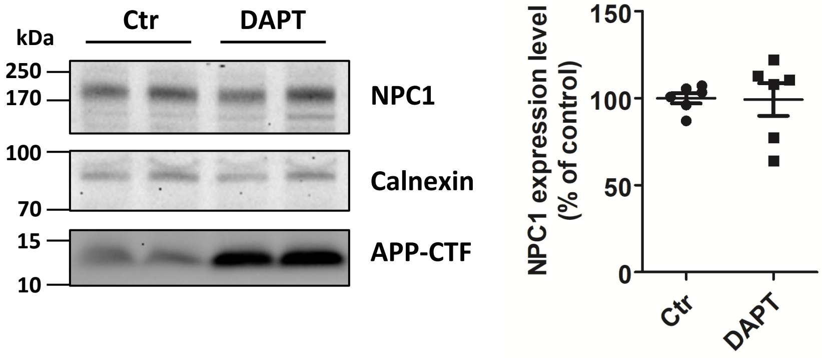

Supplementary Figure. S3. Effect of γ-secretase inhibition on NPC1 expression in wild-type mouse embryonic fibroblasts. Wild-type mouse embryonic fibroblasts (WT MEFs) were treated with (DAPT) or without (Ctr) DAPT at 10 µM. Representative western blotting pictures (left) and relative quantification of NPC1 (right). Signal intensities were normalized to the signals of calnexin. Values were obtained from three independent experiments with two biological replicates (n=3). APP-CTF; amyloid precursor protein C-terminal fragment.
